# Derivation of human trophoblast stem cells from placentas at birth

**DOI:** 10.1016/j.jbc.2025.108505

**Published:** 2025-04-10

**Authors:** Victoria Karakis, John W. Britt, Mahe Jabeen, Adriana San Miguel, Balaji M. Rao

**Affiliations:** 1 Department of Chemical and Biomolecular Engineering, North Carolina State University, Raleigh, North Carolina, USA; 2 Department of Biological Sciences, North Carolina State University, Raleigh, North Carolina, USA; 3 Golden LEAF Biomanufacturing Training and Education Center, North Carolina State University, Raleigh, North Carolina, USA

**Keywords:** human trophoblast stem cells, extravillous trophoblast, syncytiotrophoblast, cytotrophoblast, placenta

## Abstract

Human trophoblast stem cells (hTSCs) have emerged as a powerful tool for modeling the placental cytotrophoblast (CTB) *in vitro*. hTSCs were originally derived from CTBs of the first-trimester placenta or blastocyst-stage embryos in trophoblast stem cell medium (TSCM) that contains epidermal growth factor , the glycogen synthase kinase-beta inhibitor CHIR99021, the transforming growth factor-beta inhibitors A83-01 and SB431542, valproic acid, and the Rho-associated protein kinase inhibitor Y-27632. Here, we show that hTSCs can be derived from CTBs isolated from the term placenta, using TSCM supplemented with a low concentration of mitochondrial pyruvate uptake inhibitor UK5099 and lipid-rich albumin (TUA medium). Notably, hTSCs could not be derived from term CTBs using TSCM alone, or in the absence of either UK5099 or lipid-rich albumin. Strikingly, hTSCs cultured in TUA medium for a few passages could be transitioned into TSCM and cultured thereafter in TSCM. hTSCs from term CTBs cultured in TUA medium as well as those transitioned into and cultured in TSCM thereafter could be differentiated to the extravillous trophoblast and syncytiotrophoblast lineages and exhibited high transcriptome similarity with hTSCs derived from first-trimester CTBs. We anticipate that these results will enable facile derivation of hTSCs from normal and pathological placentas at birth with diverse genetic backgrounds and facilitate *in vitro* mechanistic studies in trophoblast biology.

Upon embryo implantation, the outer trophectoderm layer of the blastocyst-stage embryo gives rise to the epithelial cytotrophoblast (CTB), which forms the two major differentiated cell lineages in the placenta—the multinucleate syncytiotrophoblast (STB) and the extravillous trophoblast (EVT) (1). Dysfunctional trophoblast development leads to defects in placentation and contributes to several pregnancy-related pathologies including preeclampsia, intrauterine growth restriction, and preterm birth (2, 3). However, mechanistic studies on early human trophoblast development are challenging due to restrictions on research with human embryos and fetal tissue, limited availability of placental samples from early gestation, and significant differences in placental development in humans and other commonly used animal models (4). Therefore, there is significant interest in the development of *in vitro* models that accurately mimic human trophoblast development *in vivo*.

In this context, human trophoblast stem cells (hTSCs)—first derived by Okae *et al.* (5) from first-trimester CTBs and blastocyst-stage embryos—have emerged as a powerful *in vitro* model for studying human trophoblast development. Analogous to the placental CTB, hTSCs can be expanded in cell culture and can be differentiated to the STB and EVT lineages. In other studies, Haider *et al.* (6) and Turco *et al.* (7) derived self-renewing trophoblast organoids (TOs) from first-trimester CTBs. Unlike hTSCs that are relatively homogenous in 2D culture, TOs are 3D structures that contain both CTB-like multipotent cells and STB.

Arguably, a limitation of hTSCs and TOs derived from first-trimester placental tissues, obtained through elective termination of pregnancy, is that the associated pregnancy outcome is typically unknown. As an alternative, several studies have established hTSCs by differentiation of human embryonic stem cells (hESCs) derived from the inner cell mass of blastocyst-stage embryos or human induced pluripotent stem cells obtained by reprogramming of somatic cells by overexpression of pluripotency-associated genes (8, 9, 10). Additionally, somatic cells have been directly reprogrammed to form human induced trophoblast stem cells by overexpression of trophoblast-specific genes (11, 12). These approaches potentially enable the development of hTSC models using somatic cells associated with known normal or pathological outcomes. However, further work is needed to assess epigenetic differences between hTSC models obtained from reprogrammed somatic cells and primary placental tissue.

Placental growth continues throughout gestation, suggesting that trophoblast stem cells may be present even in placentas at term. Yet, hTSCs or TOs cannot be derived from term placentas using previously described protocols (5, 6, 7). Nevertheless, results from two recent studies suggest that hTSCs and TOs can indeed be derived from term placentas. Yang *et al.* modified previous protocols described by Turco *et al.* (7) to derive organoids from term placentas (13). Wang *et al.* have derived hTSCs from term placentas by culturing CTBs in trophoblast stem cell medium (TSCM) described by Okae *et al.*, at 1% oxygen (14).

In this study, we report the derivation and characterization of hTSCs using CTBs isolated from term placentas, in TSCM supplemented with a low concentration (50 nM) of the mitochondrial pyruvate uptake inhibitor UK5099 and AlbuMAX II, that is, lipid-rich albumin (TUA medium). Notably, our culture conditions do not require the use of hypoxic conditions. Furthermore, we show that cells cultured for a few passages in TUA medium can be transitioned to TSCM and cultured further, like hTSCs derived from first-trimester placentas. hTSCs from term placentas derived and maintained in TUA medium, as well as those transitioned into TSCM, exhibit high transcriptome similarity with hTSCs from first-trimester placentas and can be differentiated to EVT and STB *in vitro*. The ability to easily generate hTSC lines from normal and pathological placentas at birth will accelerate mechanistic studies in human placental biology.

## Results

### Derivation of hTSCs from term CTBs in TUA medium

**Figure 1:**
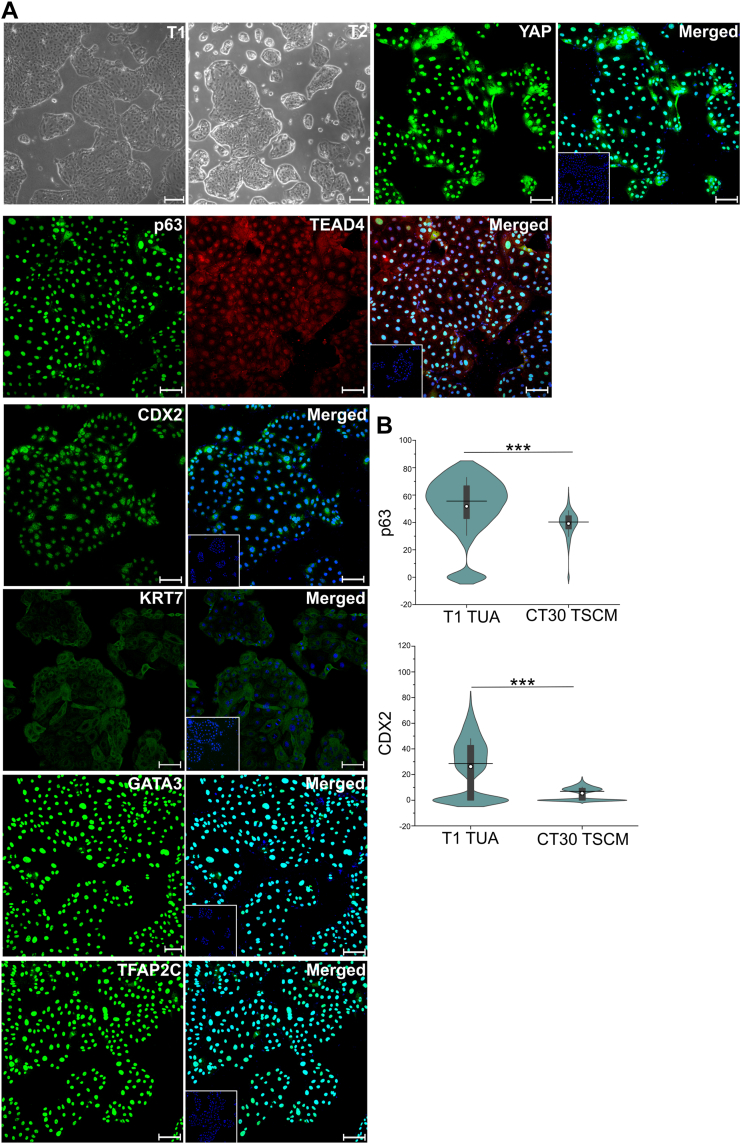
**Expression of hTSC markers in TUA medium***A*, bright field images for T1 and T2 hTSCs in TUA medium. Confocal microscopy imaging of T1 hTSCs cultured in TUA medium, staining for TFAP2C, YAP, GATA3, KRT7, TEAD4, p63, and CDX2; p63 was costained with TEAD4. Nuclei were stained with DAPI (*blue*). Isotype control is shown as an inset image (same as in Fig. S1*A*). Scale bars represent 100 μm for confocal images and 250 μm for bright field images. *B*, quantification of expression of p63 and CDX2 from T1 hTSCs (n = 5565 for p63, n = 6631 for CDX2) and CT30s hTSCs (n = 1259 for p63, n = 7813 for CDX2). Data from two biological replicates were used. *White circle* represents the mean and the *black line* represents the median (∗∗∗*p* value < 0.001). hTSC, human trophoblast stem cell.

Okae *et al.* derived hTSCs from first-trimester placentas and blastocyst-stage embryos in TSCM that contains epidermal growth factor (EGF), the glycogen synthase kinase-beta inhibitor CHIR99021, the transforming growth factor-beta inhibitors A83-01 and SB431542, valproic acid (VPA), and the Rho-associated protein kinase inhibitor Y27632. We derived two hTSC lines (T1(female) and T2 (male)) using CTBs isolated from term placentas with no known pathology using TSCM supplemented with a low concentration (50 nM) of the mitochondrial pyruvate uptake inhibitor UK5099 and lipid-rich bovine serum albumin (BSA) (AlbuMAX II, 0.2%); this medium is referred to as TUA medium. Briefly, CTBs from term placentas expressing CD49f (integrin α6) were isolated as described in Experimental procedures and cultured in TUA medium on tissue culture plates coated with vitronectin/laminin-521. Similar to observations by Okae *et al.* (5), we observed slow growth initially; however, highly proliferative cells with morphology similar to hTSCs derived from first-trimester placentas were seen within a few passages (Fig. 1*A*). Under these conditions, cells express the pan-trophoblast marker KRT7 and markers associated with hTSCs including GATA3, TFAP2C, YAP, TEAD4, and p63 (Figs. 1*A* and S1*A*). Data from CT29 and CT30 hTSCs derived from first-trimester placentas are shown for comparison in Fig. S1, *B*–*D*. Interestingly, in contrast to CT29 and CT30 hTSCs, T1 and T2 hTSCs from term placentas showed expression of the early trophoblast marker CDX2 (Figs. 1*A* and S1, *A*, *C*, and *D*). Quantitative comparisons of CDX2 and p63 expression intensity in immunofluorescence images are shown in Figures 1*B* and S1*B*. T1 and T2 hTSCs from term placentas have been maintained for 20+ passages in TUA medium, indicating robustness of these culture conditions.

**Figure 2:**
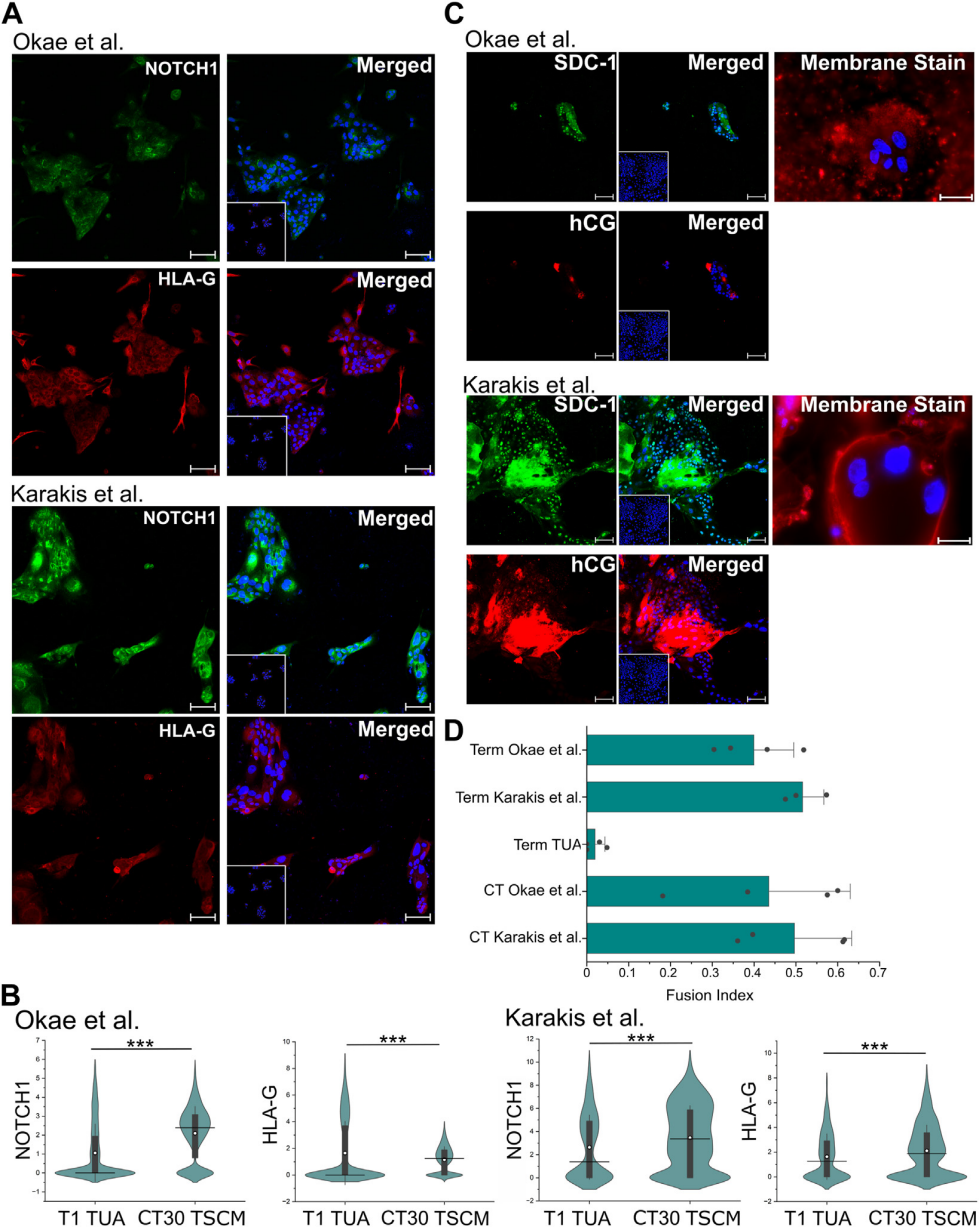
**EVT and STB differentiation of hTSCs from term CTBs in TUA medium.***A*, confocal microscopy imaging of T1 hTSCs cultured in TUA and differentiated for 6 days using protocols by Okae *et al.* or Karakis *et al.*, staining for NOTCH1 and HLA-G at day 6 of differentiation. Nuclei were stained with DAPI. Inset image is isotype control (same as in Fig. S2*B*). *B*, quantification of NOTCH1 and HLA-G expression from T1 hTSCs and CT30s differentiated with protocols by Okae *et al.* or Karakis *et al.* Data from two biological replicates were used. *White circle* represents the mean and the *black line* represents the median. For T1 hTSCs cultured in TUA using protocol by Okae *et al.*, n = 10,992; Karakis *et al.*, n = 844. For CT30 hTSCs using protocol by Okae *et al.*, n = 5036; Karakis *et al.*, n = 1436. (∗∗∗*p* value < 0.001, n.s. = not statistically significant (*p* > 0.05)). Data for EVT differentiation using protocol by Okae *et al.* have been obtained from our previously published work (15) and reanalyzed for this figure. *C*, Di-8-ANEPPS membrane staining and confocal microscopy imaging staining for hCG and SDC-1 at day 6, for T1 hTSCs differentiated to STB using protocols by Okae *et al.* or Karakis *et al.* Nuclei were stained with DAPI. Inset images are isotype control (same as in Fig. S2*E*). *D*, fusion index of STB from hTSCs from term (T1 and T2) and first-trimester (CT29 and CT30) CTBs, differentiated using protocols by Okae *et al.* or Karakis *et al.* T1 and T2 hTSCs in TUA medium are used as a control. The fusion index was calculated as (N-S)/T, where N is the number of nuclei in syncytia, S is the number of syncytia, and T is the total count of nuclei in both fused and unfused cells. Analysis was conducted using at least seven images, for each biological replicate. All syncytia had a minimum of three nuclei within them. Data for STB differentiation using protocol by Okae *et al.* have been obtained from our previously published work (15) and reanalyzed for this figure. Scale bars respresent 100 μm for all images. CTB, cytotrophoblast; EVT, extravillous trophoblast; hTSC, human trophoblast stem cell; STB, syncytiotrophoblast.

We further assessed the differentiation of T1 and T2 hTSCs derived from term CTBs to the STB and EVT lineages using chemically defined differentiation conditions previously described by Karakis *et al.* (15), or using protocols described by Okae *et al.* (5) (Fig. S2, *A* and *D*). Differentiation of CT29 and CT30 hTSCs derived from first-trimester placentas was used as a comparison (Fig. S3). With both protocols for EVT differentiation, T1 and T2 hTSCs gave rise to cells expressing the EVT markers NOTCH1 and HLA-G (Figs. 2*A* and S2*B*). Quantitative image analysis showed that protein expression levels of NOTCH1 and HLA-G were comparable between EVTs from T1 and T2 hTSCs from term placentas and CT29 and CT30 hTSCs previously derived by Okae *et al.* under both sets of differentiation conditions used (Figs. 2*B* and S2*C*).

We have previously described chemically defined conditions for STB differentiation of hTSCs in the absence of forskolin (15). Under these conditions, a membrane stain showed that T1 and T2 hTSCs formed multinucleate cells (Figs. 2*C* and S2*F*). The fusion indices for the multinucleate cells formed from T1 and T2 hTSCs were comparable to that previously reported for CT29 and CT30 hTSCs under identical differentiation conditions (Fig. 2*D*). Additionally, the differentiated cells expressed the STB markers hCG and SDC-1 (Figs. 2*C* and S2*E*). Interestingly, considerable cell death was observed when T1 and T2 hTSCs were differentiated in the presence of forskolin, using the protocol described by Okae *et al.* (5). Nevertheless, fusion indices in the surviving cells were comparable to that seen in case of CT29 and CT30 hTSCs differentiated in the presence of forskolin (Fig. 2*D*), and we observed expression of the STB markers hCG and SDC-1 (Figs. 2*C* and S2*E*).

Taken together, our results show that hTSCs can be derived from term CTBs in TUA medium. These hTSCs can be extensively passaged in TUA medium and retain the ability to differentiate to EVT and STB lineages.

### hTSCs from term placentas can be transitioned into TSCM and cultured extensively and retain differentiation potential

We observed that stable hTSC cultures could not be established from CD49f+CTBs isolated from term placentas in TSCM, even though cells could be maintained over a few passages. During these early passages, we observed expression of the pan-trophoblast marker KRT7 and markers associated with hTSCs such as GATA3, TEAD4, TFAP2C, YAP, and p63 (Fig. S4*A*). Interestingly, we also observed expression of CDX2; quantitative image analysis comparing expression levels of CDX2 and p63 relative to hTSCs from term CTBs in TUA medium is shown in Fig. S4*B*. Further, we observed that cells in TSCM at early passage numbers did not efficiently differentiate to EVT or STB lineages using protocols described by Okae *et al.* or chemically defined differentiation conditions described by Karakis *et al.*; expression of the EVT markers Notch1 and HLA-G, or the STB markers SDC-1 and hCG, were low or not observed using either set of differentiation conditions (Fig. S4, *C* and *D*). These results are consistent with the findings reported by Okae *et al.* that hTSCs could not be derived from term CTBs in TSCM (5).

**Figure 3:**
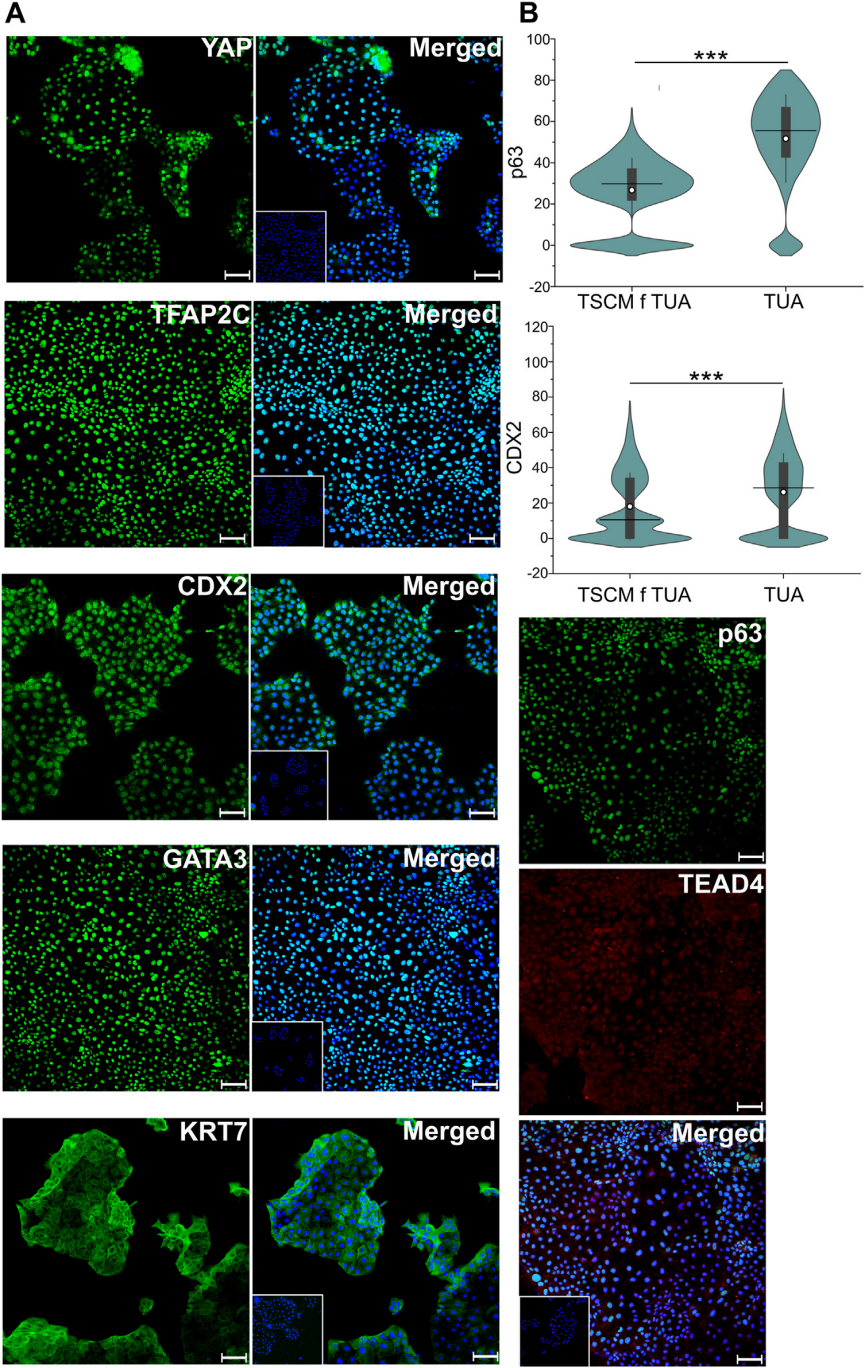
**Expression of hTSC markers in cells transitioned to TSCM.***A*, confocal microscopy imaging of T1 hTSCs transitioned to TSCM, staining for TFAP2C, YAP, GATA3, KRT7, p63, TEAD4, and CDX2. p63 was costained with TEAD4. Nuclei were stained with DAPI. Inset images are isotype control (same as in Fig. S1*A*). Scale bars represent 100 μm. *B*, quantification of expression of p63 and CDX2 from T1 hTSCs transitioned into TSCM (n = 9931 for p63, n = 9210 for CDX2). Data from two biological replicates were used; data from T1 hTSCs in TUA medium (same data as in Fig. 1) is shown for comparison. *White circle* represents the mean, and the *black line* represents the median (∗∗∗*p* value < 0.001). Scale bars represent 100 μm for all images. hTSC, human trophoblast stem cell; TSCM, trophoblast stem cell medium.

However, strikingly, we observed that T1 and T2 hTSCs cultured in TUA medium for five passages could be transitioned to TSCM and cultured for 20+ passages. In TSCM, T1 and T2 hTSCs retained expression of markers associated with hTSCs, including GATA3, TFAP2C, YAP, TEAD4, and p63 (Figs. 3*A* and S5*A*). T1 and T2 hTSCs transitioned into TSCM also retained expression of CDX2, although quantitative image analysis showed lower expression of CDX2 and p63 in TSCM relative to cells in TUA medium (Figs. 3*B* and S5*B*).

**Figure 4:**
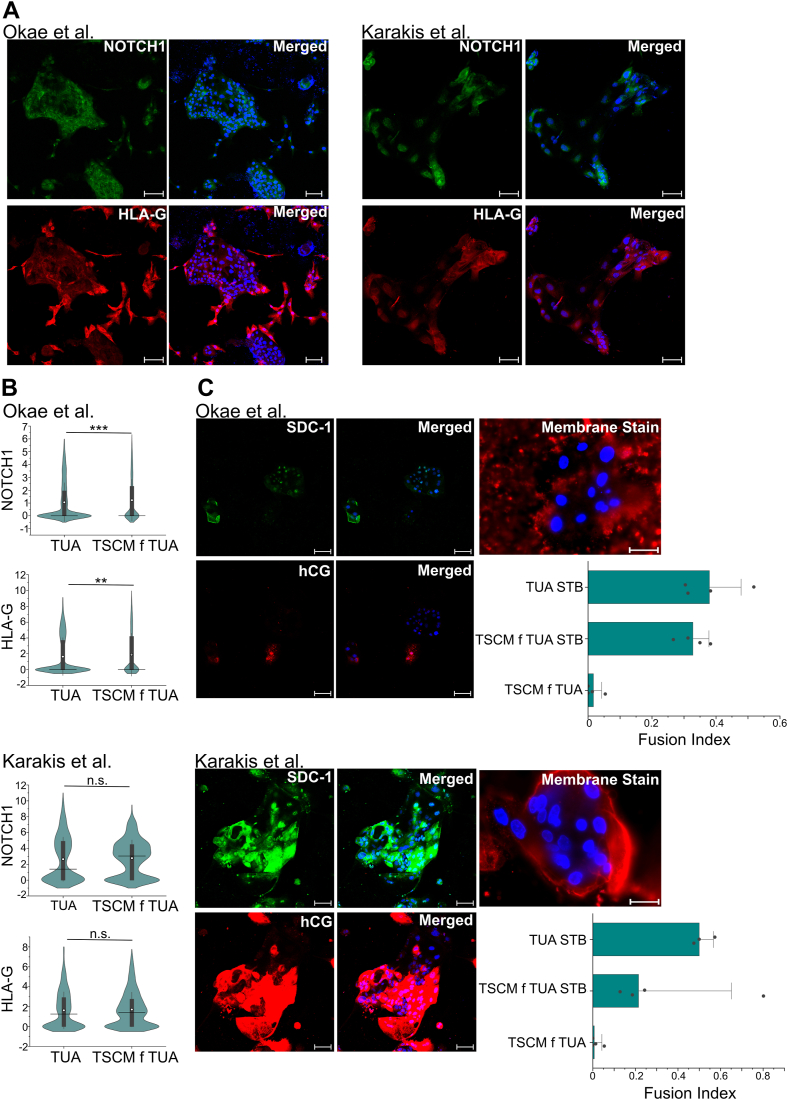
**EVT and STB differentiation of hTSCs transitioned into TSCM.***A*, confocal microscopy imaging of T1 hTSCs transitioned into TSCM and differentiated for 6 days using protocols by Okae *et al.* or Karakis *et al.*, staining for NOTCH1 and HLA-G at day 6 of differentiation. Nuclei were stained with DAPI. *B*, quantification of NOTCH1 and HLA-G expression from T1 hTSCs transitioned into TSCM and differentiated with protocols by Okae *et al.* (n = 2986) or Karakis *et al.* (n = 1080). Data from two biological replicates were used. Data from T1 hTSCs in TUA medium is shown for comparison (same data as in Fig. 2). *White circle* represents the mean and the *black line* represents the median. (∗∗*p* value < 0.01, ∗*p* value < 0.05, n.s. = not statistically significant (*p* > 0.05)). *C*, Di-8-ANEPPS membrane staining and confocal microscopy imaging staining for hCG and SDC-1 at day 6, for T1 hTSCs in TSCM, differentiated to STB using protocols by Okae *et al.* or Karakis *et al.* Nuclei were stained with DAPI. Fusion Index of STB from hTSCs from term placentas (T1 and T2) in TSCM, compared with STB from T1 and T2 hTSCs in TUA medium (same data as Fig. 2), differentiated using protocols by Okae *et al.* or Karakis *et al.* T1 and T2 hTSCs in TSCM are used as a control. The fusion index was calculated as (N-S)/T, where N is the number of nuclei in syncytia, S is the number of syncytia, and T is the total count of nuclei in both fused and unfused cells. Analysis was conducted using at least seven images, for each biological replication. All syncytia had a minimum of three nuclei within them. Scale bars represent 100 μm for all images. EVT, extravillous trophoblast; hTSC, human trophoblast stem cell; STB, syncytiotrophoblast; TSCM, trophoblast stem cell medium.

T1 and T2 hTSCs transitioned into TSCM also retained the ability to differentiate into EVTs and STB. As in the case for cells in TUA medium, we differentiated T1 and T2 hTSCs in TSCM to EVT lineage using protocols described by Okae *et al.* (5) and Karakis *et al.* (15). Differentiated cells expressing the EVT markers Notch1 and HLA-G were obtained using both protocols (Figs. 4, *A* and *B* and S6, *A* and *B*). Like the case for TUA medium, T1 and T2 hTSCs transitioned to TSCM formed multinucleate cells when differentiated under chemically defined conditions in the absence of forskolin (protocol by Karakis *et al.*) (Figs. 4*C* and S6*C*). However, forskolin-mediated STB differentiation of T1 and T2 hTSCs transitioned into TSCM, using the protocol described by Okae *et al.*, resulted in considerable cell death, like the results observed for T1 and T2 hTSCs in TUA medium. Nevertheless, we observed expression of the STB markers hCG and SDC-1 (Figs. 4*C* and S6*C*), and fusion indices in the surviving cells were comparable to that seen in case of hTSCs in TUA medium differentiated using the same protocol (Fig. 4*C*).

Taken together, these results show that even though hTSCs cannot be derived in TSCM from term CTBs, hTSCs derived in TUA medium can be transitioned into TSCM and cultured extensively thereafter. Further, hTSCs from term CTBs transitioned into TSCM retain their ability to undergo differentiation to EVT and STB lineages.

### Transcriptome analysis of hTSCs derived from term CTBs

We used genome wide RNA-Seq analysis to characterize the transcriptome of T1 and T2 hTSCs cultured in TUA medium or hTSCs transitioned into TSCM from TUA medium (denoted TSCM f TUA). Using data from the literature, we compared the transcriptomes of T1 and T2 hTSCs in these conditions, CT29 and CT30 hTSCs cultured in TSCM (8), hTSCs derived from term CTB in TSCM at 1% oxygen concentration by Wang *et al.* (14), and primary villous CTBs (vCTBs) isolated from the first-trimester (6); CT29 and CT30 hTSCs were used as the control group for comparison in all analysis. A comprehensive list of differentially expressed genes (DEGs) (|log_2_ fold change|≥1) is included in Tables S1–S3.

**Figure 5:**
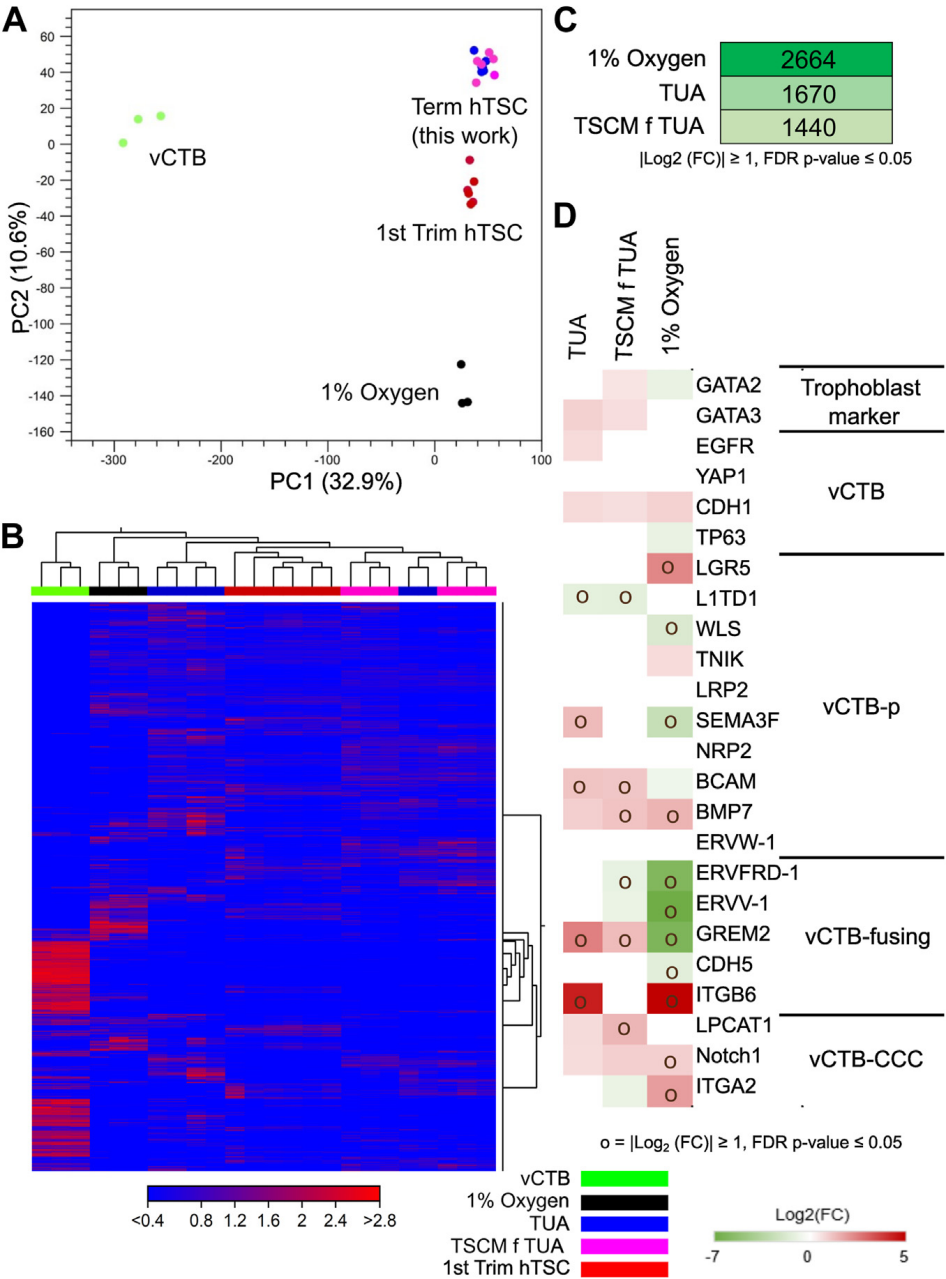
**Transcriptome analysis of hTSCs derived from term CTBs.***A*, principal component analysis (PCA) of transcriptome data from primary villous CTB (vCTB), CT29, and CT30 hTSCs cultured in TSCM (first-trimester hTSC), T1 and T2 hTSCs from term CTBs cultured in TUA medium and TSCM f TUA (term hTSC), and hTSCs from term CTB derived in TSCM at 1% oxygen (1% oxygen). *B*, hierarchical clustering analysis of transcriptome data for the same samples used in PCA analysis. *C*, number of differentially expressed genes (DEGs) for term hTSCs in TUA medium and TSCM f TUA, and hTSCs derived from term CTBs under 1% oxygen, with false discovery rate (FDR) *p* value < 0.05 and |log_2_ (fold change) (FC)| ≥ 1. First-trimester hTSCs (CT29, CT30) were used as the control group. *D*, transcript expression for a targeted panel of genes associated with vCTB or early vCTB differentiation; relative expression of mRNA compared to first-trimester hTSCs. “o” indicates relative expression levels where |log_2_ (fold change) (FC)| ≥ 1 and FDR *p* value < 0.05. *Blank grids* indicate FDR *p* value> 0.05, *that is,* not significant. CTB, cytotrophoblast; hTSC, human trophoblast stem cell; TSCM, trophoblast stem cell medium; vCTB-CCC, vCTB–cytotrophoblast cell column; vCTB-p, proliferative vCTB.

Principal component analysis (PCA) (Fig. 5*A*) and hierarchical clustering analysis (Fig. 5*B*) showed high transcriptome similarity between T1 and T2 hTSCs in TUA or TSCM f TUA and CT29 and CT30 hTSCs, although both have differences relative to primary vCTBs from the first-trimester. On the other hand, hierarchical clustering analysis revealed distinct differences between hTSCs derived from term CTBs at 1% oxygen and the other hTSC samples. Notably, 2664 genes are differentially regulated in hTSCs derived at 1% oxygen relative to CT29 and CT30 hTSCs, as compared to 1670 and 1440 genes for T1 and T2 hTSCs in TUA medium and TSCM f TUA, respectively (Fig. 5*C*). We further investigated a targeted panel of genes associated with vCTB or vCTB undergoing early differentiation as described (16) (Fig. 5*D*). We did not observe substantial differences in transcript expression of these genes between T1 and T2 hTSCs in TUA and TSCM f TUA relative to CT29 and CT30 hTSCs, with similar expression of the established trophoblast and vCTB markers *GATA2*, *GATA3*, *EGFR*, *YAP1*, *CDH1*, and *TP63* (|log_2_ fold change| < 1). However, there were some differences observed in expression of markers associated with proliferative trophoblasts or markers associated with fusing vCTBs (vCTB-fusing) or CTB cell columns; nevertheless, greater differences were observed in hTSCs derived under 1% oxygen.

**Figure 6:**
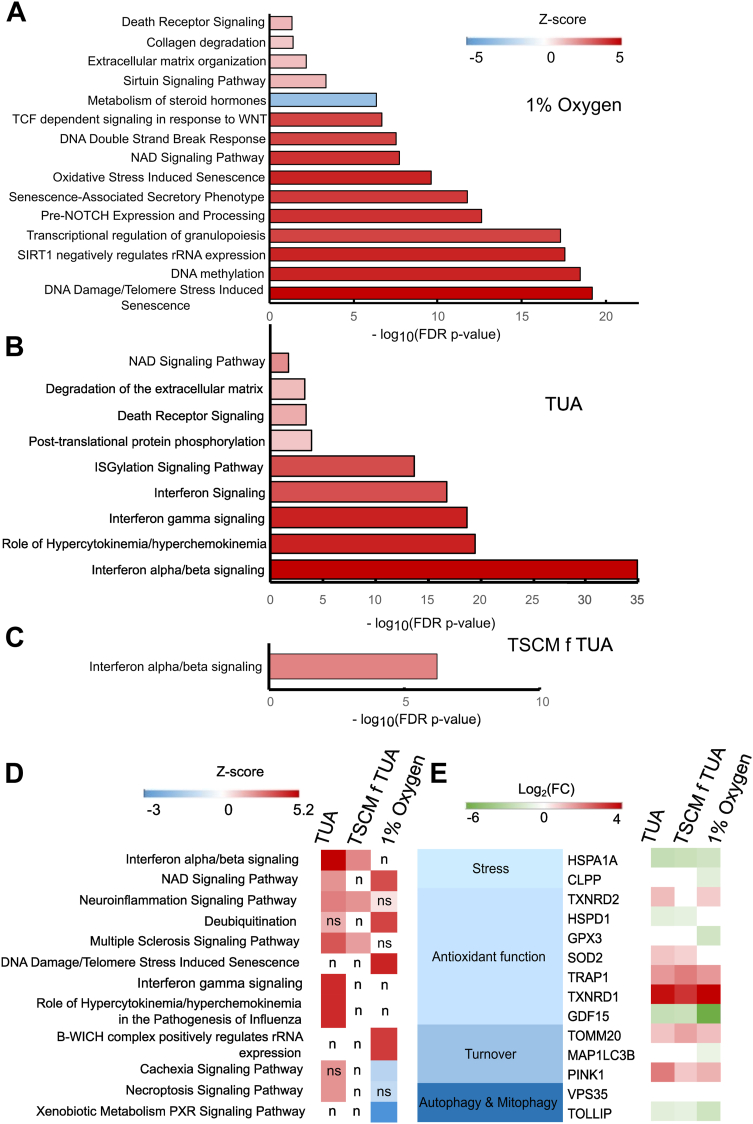
**Ingenuity pathway analysis of most abundant genes in term hTSCs**. *A*–*C*, IPA for hTSCs from term CTBs derived under 1% oxygen (*A*) and T1 and T2 hTSCs in TUA medium (*B*) and TSCM f TUA (*C*) first-trimester hTSCs (CT29, CT30) were used as the control group. IPA includes the top five canonical pathways and other significant trophoblast-related cellular pathways. Most abundant genes were selected following the cutoff FDR *p* value ≤0.05, |log2 (fold change (FC))| ≥ 2, maximum group mean ≥ 5. *D*, comparative IPA pathway analysis between hTSCs from term CTBs. First-trimester hTSCs (CT29, CT30) were used as the control group. IPA includes the top canonical pathways. Most abundant genes were selected following the cutoff FDR *p* value ≤0.05, |log2 (fold change (FC))| ≥ 2, maximum group mean ≥ 5. Here, n indicates Z-score is not available for that comparison, ns indicates FDR *p* value> 0.05, *that is,* nonsignificant. *E*, targeted panel of mitochondrial genes showing differences in relative mRNA expression between hTSCs from term CTBs and hTSCs from first-trimester CTBs, with FDR *p* value ≤0.05. *Blank grids* indicate FDR *p* value> 0.05, *that is,* nonsignificant. CTB, cytotrophoblast; FDR, false discovery rate; hTSC, human trophoblast stem cell; IPA, Ingenuity pathway analysis.

We further used ingenuity pathway analysis (IPA) to assess pathways that are differentially regulated in T1 and T2 hTSCs in TUA, TSCM f TUA, and hTSCs from term CTBs derived under 1% oxygen, relative to CT29 and CT30 hTSCs (Fig. 6, *A*–*D* and Tables S4–S6). IPA shows that hTSCs derived in 1% oxygen exhibit significant upregulation of DNA damage–induced and oxidation stress–induced senescence pathways (Fig. 6*A*). Further, Wnt and the pre-NOTCH expression and processing pathway, which activates NOTCH signaling downstream, were upregulated in these cells relative to CT29 and CT30 hTSCs. Notably NOTCH signaling is a key determinant of differentiation to the EVT lineage and markers for CTB cell columns are upregulated in hTSCs derived in 1% oxygen (Fig. 5*D*). Interestingly, these pathways were not upregulated in T1 and T2 hTSCs (Fig. 6, *B* and *C*); rather, we observed upregulation of interferon alpha/beta signaling in hTSCs in both TUA medium and TSCM f TUA. Additionally, interferon, interferon gamma, and ISGylation signaling pathways were upregulated in hTSCs in TUA medium but not in TSCM f TUA. Notably, the ISGylation pathway is associated with lipid metabolism (17, 18, 19, 20). Use of lipid-rich albumin is critical to deriving hTSCs from term CTBs, as discussed in the following section, and these lipids are withdrawn when hTSCs are transitioned to TSCM from TUA medium.

To provide a comprehensive comparison of upregulated and downregulated pathways, we conducted a comparative analysis of IPA outputs from Figure 6, *A*–*C* across term CTB derived hTSCs (Fig. 6*D*). Additionally, we performed cluster analysis on the DEGs identified in Figure 5*B* using the elbow method to define clusters based on their expression patterns under different derivation conditions. These clusters were then subjected to gene ontology (GO) analysis to identify enriched biological pathways. Performing GO analysis on these individual clusters allowed for more specific enrichment results; these are presented in Tables S7–S19.

Finally, as discussed in the following section, use of the mitochondrial pyruvate uptake inhibitor UK5099 was essential for derivation of functional hTSCs from term CTBs. Therefore, we investigated a targeted panel of genes associated with mitochondrial regulation (Fig. 6*E*). Specifically, we focused on a set of genes that have been reported to be differentially expressed in primary chorionic tissue in the third- trimester relative to the first-trimester (21). This previous study reported that expression of the antioxidant genes *CLPP*, *TXNRD2*, *HSPD1*, and *GPX3* was lower in tissue from the third-trimester relative to the first-trimester; on the other hand, *SOD2*, *HSPA1A*, *TRAP1*, *TXNRD1*, and *GDF15* were upregulated in tissue from the third-trimester. Additionally, expression of the mitophagy and autophagy-related genes *TOMM20*, *MAP1LC3B*, and *PINK1* was upregulated in tissue from the third-trimester, and *VPS35* and *TOLLIP* expression was downregulated. Our analysis showed that differential expression of these genes was fairly consistent across hTSCs derived from third-trimester CTBs (T1 and T2 hTSCs in TUA medium and TSCM f TUA, and hTSCs derived under 1% oxygen), relative to CT29 and CT30 hTSCs derived from first-trimester placentas. However, trends observed in third *versus* first-trimester primary tissue were not entirely conserved. For instance, similar to primary tissue, *HSPD1* and *TOLLIP* were downregulated and *SOD2*, *TRAP1*, *TXRND1*, *TOMM20*, and *PINK1* were upregulated in T1 and T2 hTSCs relative to CT29 and CT30 hTSCs. On the other hand, unlike primary tissue from third *versus* first-trimester, T1 and T2 hTSCs showed downregulation of *HSPA1A* and *GDF15*; there were no statistically significant differences in expression of *CLPP*, *GPX3*, *MAP1LC3B*, and *VPS35* between T1 and T2 hTSCs from term CTBs and CT29 and CT30 hTSCs.

Overall, our transcriptome analysis shows that T1 and T2 hTSCs derived from term CTBs, in both TUA medium and TSCM f TUA, show high transcriptome similarity to CT29 and CT30 hTSCs derived from first-trimester placentas. Nevertheless, there are some differences in transcript expression, albeit fewer than those observed for hTSCs derived from term CTBs under 1% oxygen. Collectively with data on extended culture of T1 and T2 hTSCs in TUA and TSCM f TUA (Figs. S1, S3, and S5) and differentiation to EVT and STB lineages (Figs. 2, 4, S2, and S6), our transcriptome analysis confirms that hTSCs can indeed be derived from term CTBs under atmospheric oxygen conditions using TUA medium. These hTSCs can be cultured extensively in TUA medium and subsequently in TSCM, while retaining differentiation potential.

### Comparative transcriptome analysis of hTSCs derived from term CTBs and hTSCs from human pluripotent stem cells

**Figure 7:**
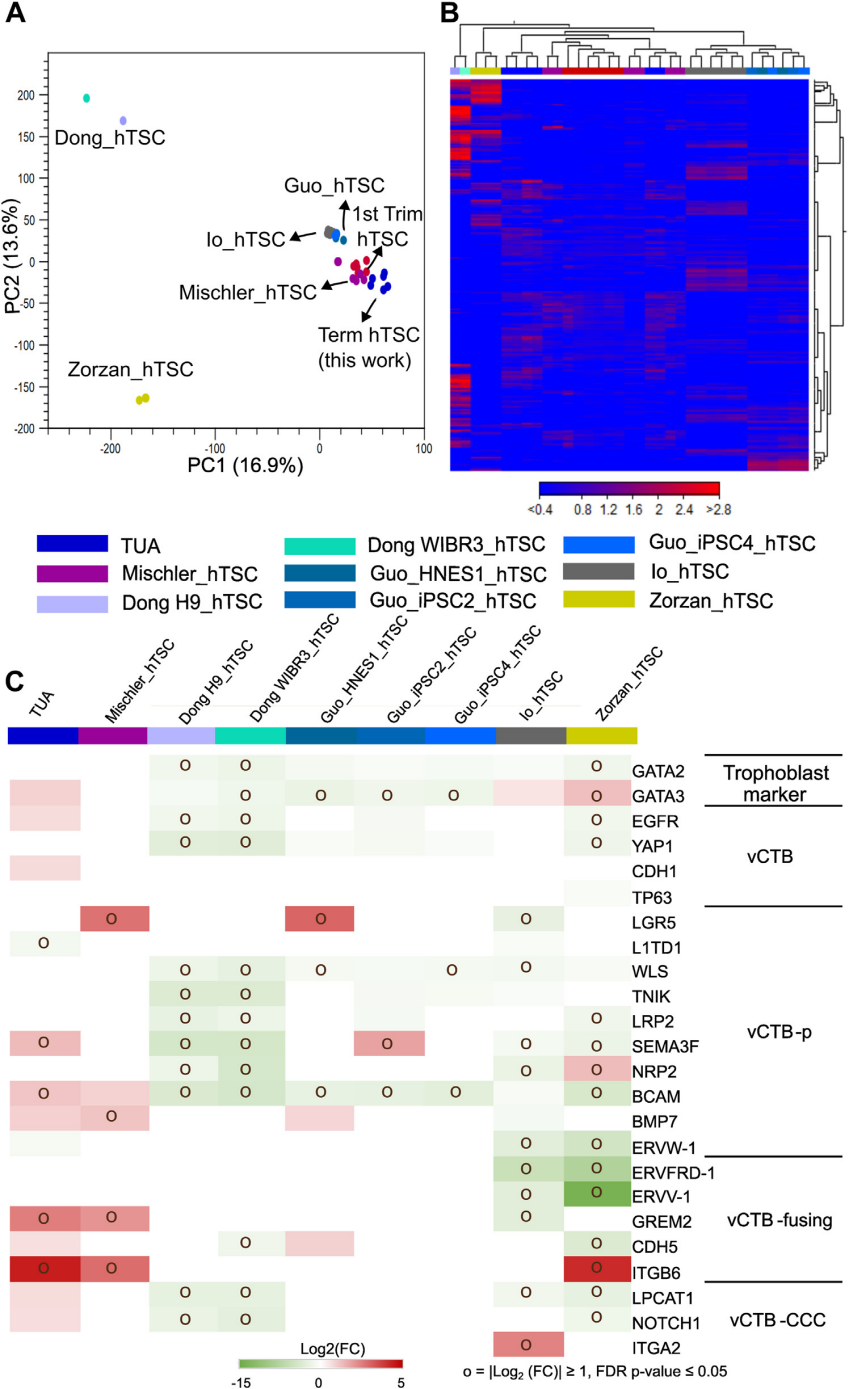
**Comparison of transcriptome analysis of hTSCs derived from term CTBs and hTSCs derived from hPSCs.***A*, principal component analysis (PCA) of transcriptome data from T1 and T2 hTSCs from term CTBs cultured in TUA medium, CT29 and CT30 hTSCs cultured in TSCM (first-trimester hTSC), and hTSCs derived from hPSCs. *B*, hierarchical clustering analysis of transcriptome data used in PCA analysis. *C*, transcript expression for a targeted panel of genes associated with vCTB or early vCTB differentiation; relative expression of mRNA compared to first-trimester hTSCs. “o” indicates relative expression levels where |log2 (fold change) (FC)| ≥ 1 and FDR *p* value < 0.05. *Blank grids* indicate FDR *p* value> 0.05, *that is,* not significant. CTB, cytotrophoblast; FDR, false discovery rate; hPSC, human pluripotent stem cell; hTSC, human trophoblast stem cell; TSCM, trophoblast stem cell medium; vCTB-CCC, vCTB–cytotrophoblast cell column; vCTB-p, proliferative vCTB.

Several studies have investigated the derivation of hTSCs from human pluripotent stem cells (hPSCs) through reprogramming or differentiation. For instance, Dong *et al.* (9) derived hTSCs from naïve hPSCs and induced pluripotent stem cells (iPSCs) (in 5i/L/A or PXGL media) by transitioning these naïve cells into TSCM. Guo *et al.* (22) generated hTSCs from hPSCs by inducing trophectoderm differentiation using PD0325901 (MEK inhibitor) and A83-01 (transforming growth factor-beta/ALK4/5/7 inhibitor) and subsequently culturing the cells in TSCM. Io *et al.* (23) developed a protocol for naive CTB-like cells from naive hPSCs *via* an intermediate naive trophectoderm-like state, followed by expansion of hTSCs in ACE medium. Zorzan *et al.* (24) established chemically reset TSCs from primed iPSCs through a resetting phase by culture in MEK inhibitor/Leukemia inhibitory factor/Histone Deacetylation inhibitor followed by PXGL medium, before transitioning to TSCM. Finally, in previous work we have shown that treatment of hPSCs with BMP4, S1P, or S1PR3 agonist and SB431542 was sufficient to generate CTB-like cells which could be subsequently transitioned to TSCM or TM4 medium to maintain two distinct hTSC populations (8). We compared hTSCs in TUA medium derived from term placentas (this study) with hTSCs derived in these aforementioned studies. Figure 7 shows a comparative transcriptome analysis using publicly available datasets from these studies (8, 9, 22, 23, 24), including PCA analysis (Fig. 7*A*), heat map analysis (Fig. 7*B*), and trophoblast-specific DEGs (Fig. 7*C*). For this analysis, we included a subset of hTSC cell lines generated in these studies—hTSCs derived from naïve H9 and WIBR3 hPSCs from Dong *et al.* (GSE138762) (9); hTSCs derived from hPSC lines HNES1, niPSC2, and niPSC4 lines by Guo *et al.* (GSE167089) (22); H9 hESC-derived naive CTB-like cells that go through an intermediate nTE state from Io *et al.* (GSE144994) (23); ccTSC.02 derived from chemically reset primed iPSCs from Zorzan *et al.* (GSE184562) (24); and hTSCs in TSCM obtained from H1 and H9 hESCs after BMP4 treatment, from Mischler *et al.* (GSE137295) (8). Unless otherwise specified, first-trimester hTSCs, CT 29 and CT 30 were used as the control group for analysis.

PCA analysis (Fig. 7*A*) reveals that term hTSCs in TUA medium cluster closely with hTSCs derived in studies by Guo *et al.* (22), Io *et al.* (23), Mischler *et al.* (8) and first-trimester hTSCs derived by Okae *et al.* (5) In contrast, hTSCs derived by Dong *et al.* and Zorzan *et al.* form distinct clusters, suggesting transcriptomic differences between these cells and term hTSCs in TUA medium. A similar clustering pattern is observed in the heatmap analysis in Figure 7*B*. Further, differential gene expression analysis (Fig. 7*C*) shows the expression patterns of key trophoblast-specific markers of vCTB or trophoblast subtypes representative of early vCTB differentiation as described (16), relative to first-trimester hTSCs. There is reasonable similarity between term hTSCs in TUA medium and hTSCs derived from hPSCs. A comprehensive list of DEGs (|log_2_ fold change|≥1) from this analysis is included in Tables S20–S27.

Collectively, these analyses show that term hTSCs in TUA medium (and term hTSCs in TSCM f TUA) exhibit transcriptional similarity with both first-trimester hTSCs and hTSCs derived from hPSCs.

### Lipid-rich albumin and UK5099 are essential for derivation of hTSCs from term CTBs

**Figure 8:**
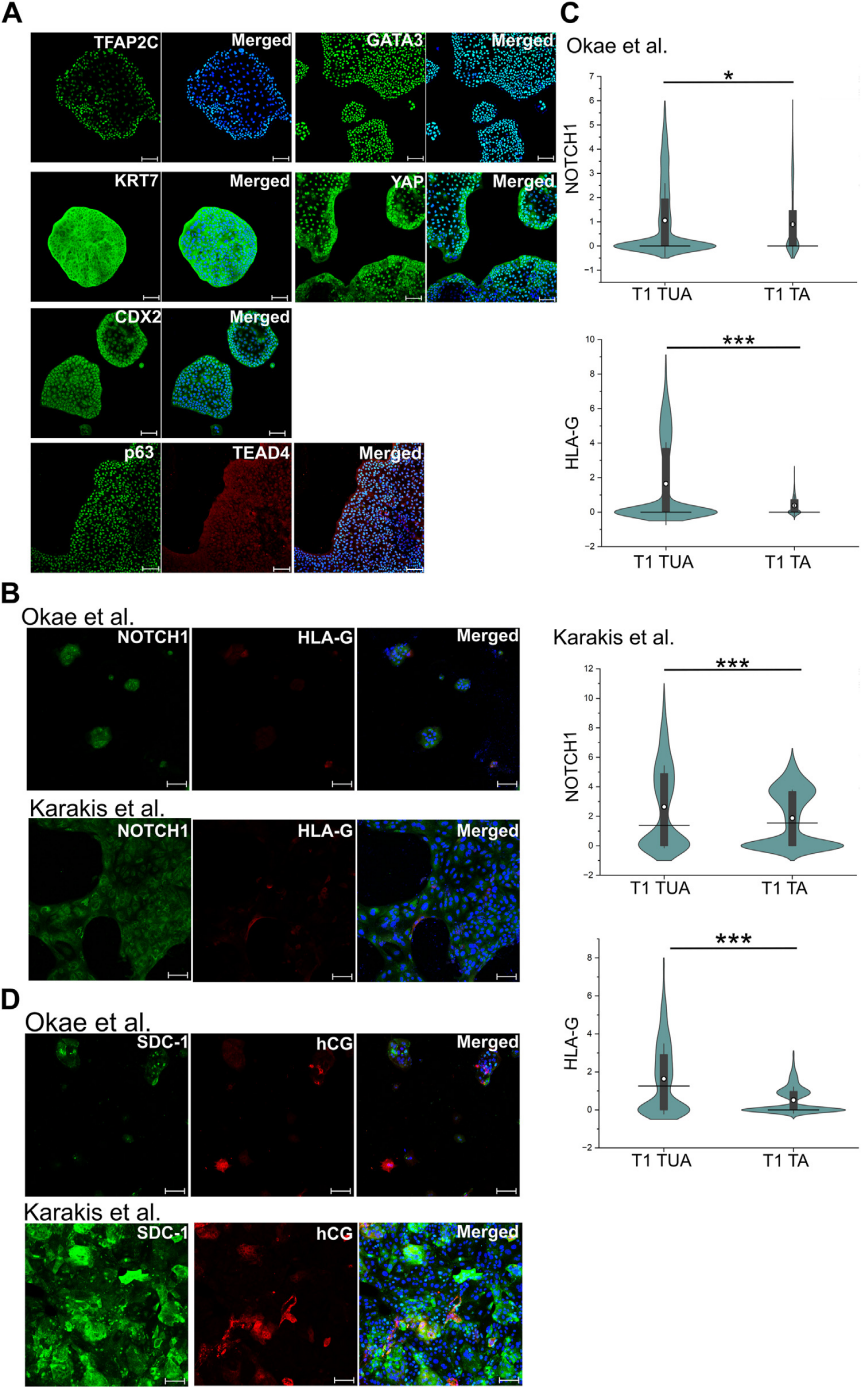
**Attempted derivation of hTSCs from term CTBs in TA medium.***A*, confocal microscopy imaging of cells obtained during attempted derivation of hTSCs in TA medium, staining for TFAP2C, YAP, GATA3, KRT7, p63, TEAD4, and CDX2; p63 was costained with TEAD4. Nuclei were stained with DAPI. Primary CTBs are from the same placenta as those used for deriving T1 hTSCs in TUA medium. *B*, confocal microscopy imaging of cells obtained during attempted derivation of hTSCs in TA medium, differentiated to EVTs for 6 days using protocols by Okae *et al.* or Karakis *et al.*, staining for NOTCH1 and HLA-G. Nuclei were stained with DAPI (*blue*). *C*, quantification of NOTCH1 and HLA-G expression in cells obtained during attempted derivation of term CTBs in TA medium (labeled T1 TA), differentiated with protocols by Okae *et al.* (n = 1356) or Karakis *et al.* (n = 1688). Data from two biological replicates were used. Data from T1 hTSCs in TUA medium are shown for comparison (same data as in Fig. 2). *White circle* represents the mean and the *black line* represents the median. (∗∗∗*p* value < 0.001, n.s. = not statistically significant (*p* > 0.05)). *D*, confocal microscopy imaging of cells obtained during attempted derivation of hTSCs in TA medium, differentiated to STB for 6 days using protocols by Okae *et al.* or Karakis *et al.*, staining for SDC-1 and hCG. Nuclei were stained with DAPI (*blue*). CTB, cytotrophoblast; EVT, extravillous trophoblast; hTSC, human trophoblast stem cell.

We observed that primary CD49f+CTBs could not be maintained in culture in TSCM supplemented with UK5099 (without AlbuMAX II) during early passages, suggesting that the presence of lipid-rich albumin is essential for establishing hTSC cultures from term CTBs. We further investigated if hTSCs can be derived from term CTBs in TSCM supplemented with AlbuMAX II alone (no UK5099); note that the same primary cells used to derive T1 and T2 hTSCs in TUA medium were used in these studies. We observed that cells could be maintained for several passages under these conditions (TA medium) and expressed relevant CTB markers including, GATA3, P63, CDX2, TFAP2C, KRT7, and YAP (Figs. 8*A* and S7*A*), like hTSCs cultured in TUA medium. However, cells in TA medium were unable to efficiently differentiate to EVT and STB using either the chemically defined conditions described by Karakis *et al.* (15) or protocols described by Okae *et al.* (5). HLA-G+EVT cells were not obtained using either protocol in T1 hTSCs; some HLA-G+ cells were observed in T2 hTSCs when the protocol by Okae *et al.* was used (Figs. 8, *B* and *C* and S7, *B* and *C*). Similarly, we did not observe significant and consistent expression of the STB markers hCG and SDC-1 (Figs. 8*D* and S7*D*). These results show that although hTSC-like cells derived from term CTBs can be maintained in TA medium, they are deficient in their ability to differentiate to EVT and STB.

**Figure 9:**
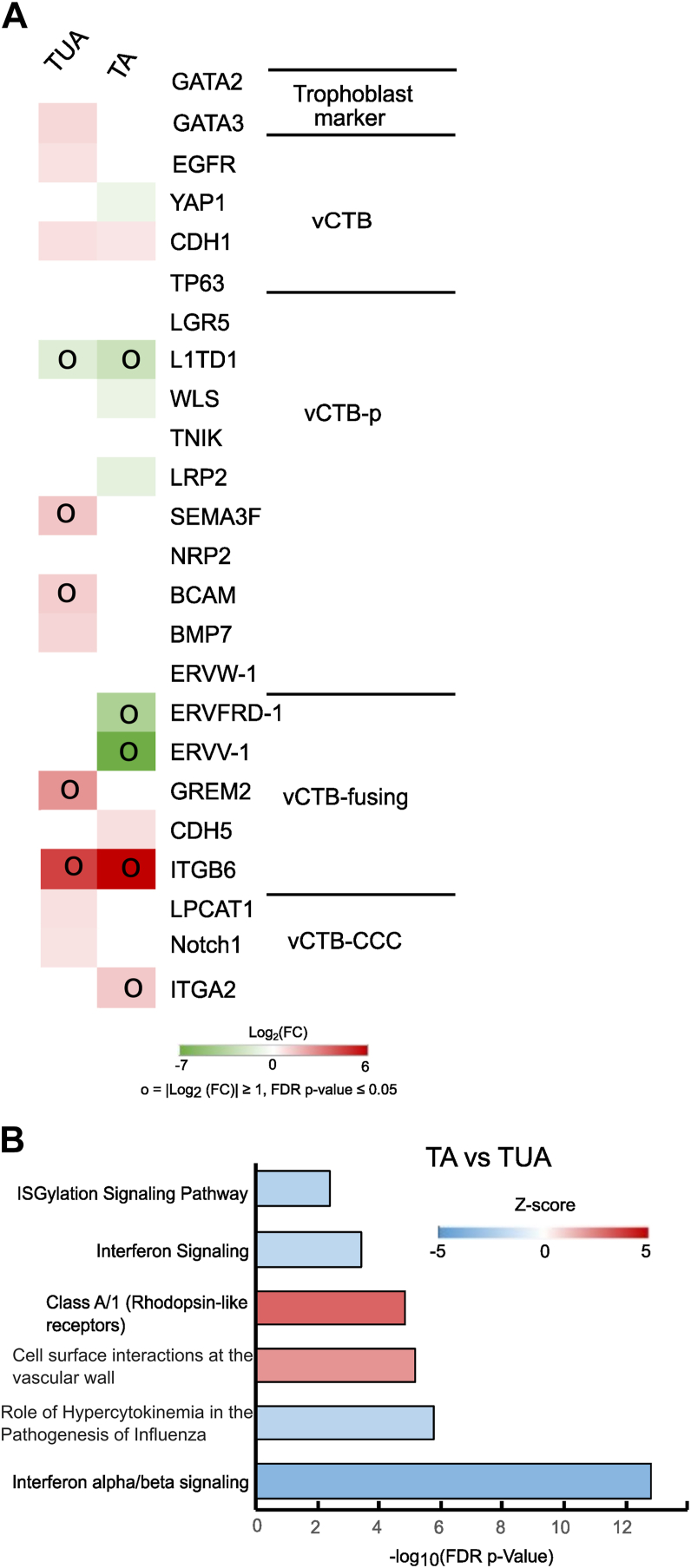
**Transcriptome analysis of hTSCs derived from term CTBs in TA.***A*, transcript expression for a targeted panel of genes associated with vCTB or early vCTB differentiation in T1 and T2 hTSCs in TUA medium or cells obtained during attempted derivation of hTSCs in TA medium; relative expression of mRNA compared to first-trimester hTSCs (CT29, CT30). “o*”* indicates relative expression levels where |log2 (fold change (FC))| ≥ 1 and FDR *p* value < 0.05. *Blank grids* indicate FDR *p* value> 0.05, that is, nonsignificant. *B*, IPA for cells obtained during attempted derivation of hTSCs in TA medium; primary CTBs are from the same placentas used for derivation of T1 and T2 hTSCs. T1 and T2 hTSCs cultured in TUA medium were used as the control group. IPA includes the top five canonical pathways and other significant trophoblast-related cellular pathways. Most abundant genes were selected for IPA with a more stringent dataset following the cutoff FDR *p* value ≤0.05, |log2 (fold change (FC))| ≥ 2, maximum group mean ≥ 5. CTB, cytotrophoblast; FDR, false discovery rate; hTSC, human trophoblast stem cell; IPA, ingenuity pathway analysis; vCTB, villous CTB.

To further examine the role of UK5099 in hTSC derivation from term CTBs, we used RNA-Seq analysis to compare the transcriptome of hTSC-like cells in TA medium and T1 and T2 hTSCs in TUA medium. The comprehensive list of differentially regulated genes is included in Table S28. In particular, we focused on the targeted panel of genes associated with the vCTB or vCTB early differentiation as described (16) (Fig. 9*A*). We observed that genes associated with proliferative vCTB are decreased in hTSC-like cells in TA medium. Nevertheless, we see high transcriptome similarity between cells in TA and TUA medium. We also used IPA to identify signaling pathways that are differentially regulated in hTSC-like cells in TA medium relative to hTSCs in TUA medium (Table S29). Interestingly, removal of the mitochondrial pyruvate uptake inhibitor UK5099 from TUA medium resulted in downregulation of ISGylation, interferon, and interferon alpha/beta signaling pathways (Fig. 9*B*), suggesting a role for mitochondrial metabolism in upregulation of these pathways in TUA medium.

Previous studies have reported that lipid-rich albumin contains phospholipids such as lysophosphatidic acid (LPA) and free fatty acids (25). We further investigated if AlbuMAX II could be replaced by LPA or CDL. Accordingly, we tried to derive hTSCs in TSCM supplemented with UK5099 and LPA (TUL medium) or CDL (TUC medium). Culture of cells in these media was far less robust than in TUA medium, limiting their analysis; indeed, expansion of cells frozen after culture of cells in TUC medium for few passages was difficult. Nevertheless, our analysis showed that cells expressing the hTSC markers GATA3 and CDX2 could be cultured in TUL medium, although they expressed lower levels of TEAD4 and exhibited greater nonnuclear staining of p63 and YAP (Fig. S8, *A* and *B*). Similarly, cells expressed CDX2 in TUC medium but showed low expression of GATA3, TEAD4, TFAP2C, and p63 (Fig. S8, *C* and *D*). However, cells in both TUL and TUC medium showed deficient differentiation to EVT and STB using two different differentiation protocols (Fig. S9); we did not consistently observe cells with the EVT markers HLA-G and Notch1 or the STB markers hCG and SDC-1. Thus, functional hTSCs could not be derived when the lipid-rich albumin in TUA medium was replaced by LPA or CDL.

Taken together, our results show that lipid-rich albumin is critical for culture and expansion of term CTBs. However, addition of the mitochondrial pyruvate uptake inhibitor UK5099 is necessary for deriving hTSCs that retain differentiation potential.

## Discussion

In this study, we have shown that hTSCs can be derived from term CTBs under atmospheric oxygen conditions in TUA medium; TUA medium is TSCM used for derivation of hTSCs from first-trimester placentas and blastocyst-stage embryos described by Okae *et al.*, supplemented with lipid-rich albumin (AlbuMAX II) and a low concentration (50 nM) of the mitochondrial pyruvate uptake inhibitor UK5099. We derived two hTSC lines, T1 (female) and T2 (male) from CD49f+CTBs isolated from term placentas with no known pathology. These hTSCs in TUA medium express markers associated with undifferentiated hTSCs and can differentiate to EVT and STB. Strikingly, hTSCs from term CTBs cultured in TUA medium for a few passages can be transitioned into TSCM and extensively cultured further. Importantly, hTSCs transitioned from TUA medium into TSCM retain their ability to differentiate to EVT and STB. Finally, hTSCs from term CTBs in TUA medium as well those transitioned into TSCM exhibit high transcriptome similarity to hTSCs derived from first-trimester CTBs.

### Role of lipid-rich albumin

TSCM contains 0.2% fetal bovine serum and 0.3% fatty acid–free BSA. Although term CTBs could be cultured for a few passages in TSCM under atmospheric oxygen conditions, they could not be maintained in long-term culture. However, cells could be extensively cultured in TSCM supplemented with lipid-rich albumin (TA medium), although these cells were deficient in differentiation potential. Strikingly, Yang *et al.* (13) modified the medium described by Turco *et al.* (7) by supplementation with 10% fetal bovine serum (FBS) for derivation of TOs from term placentas; FBS contains lipid-rich albumin. Collectively, these results suggest that lipid supplementation plays an important role in enabling derivation of self-renewing cultures (hTSCs or organoids) from late gestation placentas. In this context, Wang *et al.* have derived hTSCs from term placentas in TSCM—without additional lipid supplementation—at 1% oxygen (14); notably, hypoxia has been reported to upregulate lipid synthesis (26). Yet, it is important to note that lipid supplementation can be withdrawn after a few passages of hTSC derivation in TUA medium; hTSCs can subsequently be cultured in TSCM. Therefore, we speculate that lipid-rich albumin is necessary for initial adaptation of term CTBs—but not CTBs from early gestation—in culture and to overcome stress associated with transfer of primary cells to an *in vitro* cell culture environment. Consistent with this speculation, lipid supplementation has been shown to increase the efficiency of primary and secondary intestinal organoid formation from old mice (27).

### Role of mitochondrial pyruvate uptake inhibitor

Although hTSC-like cells can be derived from term CTBs in TSCM supplemented with lipid-rich albumin, these cells are deficient in their ability to differentiate to EVT and STB. Thus, supplementation with the mitochondrial pyruvate uptake inhibitor UK5099 is critical for derivation of hTSCs from term CTBs in our conditions. We have used a low concentration of UK5099 in our study (50 nM; IC50 is 50 nM); we observe significant cell death when high concentrations of UK5099 (greater than 10× IC_50_) are used with hTSCs in TSCM. Notably, at concentrations similar to that used here, UK5099 has been shown to increase mitochondrial respiration through lipid cycling in brown adipocytes (28). Our transcriptome analysis also shows that interferon-related signaling pathways, including ISGylation, are upregulated in TUA medium relative to TA medium. Pertinently, reduction in levels of ISG15 that is required for ISGylation is associated with preeclampsia and knockdown of ISG15 expression impairs invasion of HTR8/SVneo cells in culture (29). ISGylation has also been reported to modulate the stability of multiple proteins, including YAP, P63, and HIF1α, which play a critical role in trophoblast development (30). ISGylation is also important to maintain efficient oxidative phosphorylation in pancreatic cancer stem cells (19). Further studies are needed to identify the specific molecular mechanisms through which UK5099 exerts its effects on hTSCs in TUA medium.

### Comparison of hTSCs from term placentas with hTSCs from early gestation

Our analysis shows high transcriptome similarity between hTSCs from term CTBs derived in TUA medium and hTSCs (CT29, CT30) from early gestation. Further, hTSCs in TUA medium can be transitioned into the same medium used for culture of hTSCs from early gestation. Like hTSCs in TUA medium, the transitioned hTSCs in TSCM exhibit high transcriptome similarity to early gestation hTSCs and retain the ability to differentiate into EVT and STB. Moreover, T1 and T2 hTSCs derived in this study showed protein expression of the early trophoblast marker CDX2, unlike CT29 and CT30 hTSCs although upregulation of *CDX2* transcript was not found to be statistically significant in RNA-Seq analysis (log_2_ fold change = 1.29 for comparison between T1 and T2 hTSCs *versus* CT29 and CT30 hTSCs, false discovery rate (FDR) *p* value = 0.14). These results are consistent with observations by Wang *et al.* who report high transcriptome similarity between hTSCs derived at 1% oxygen and hTSCs from early gestation (14). Similarly, Yang *et al.* show that the transcriptome of TOs derived from term placentas is similar to first-trimester placental tissue (13). Strikingly, we also observe transcriptome similarity between T1 and T2 hTSCs and hTSCs derived from hPSCs in several different studies (Fig. 7). Collectively, these studies raise the intriguing possibility that derivation of hTSCs from term CTB results in reprogramming to an earlier developmental state. Such reprogramming is likely dependent on epigenetic modifications upon culture of term CTBs. Indeed, VPA, a known epigenetic modifier, is a key component of TSCM. Further studies are needed to rigorously assess epigenetic differences between hTSCs derived from term placentas, hTSCs from early gestation, and primary CTBs across gestation.

In conclusion, we have shown that functional hTSCs can be derived in a straightforward manner from placentas at birth using TUA medium, that is, TSCM supplemented with UK5099 and AlbuMAX II. Importantly, our approach does not require equipment for control of oxygen concentrations and once derived, hTSCs can be transitioned into TSCM that is widely used for culture of hTSCs from first-trimester placentas and blastocyst-stage embryos. These results will enable facile derivation of hTSCs from normal and pathological placentas at birth and enable mechanistic studies that address critical knowledge gaps in our understanding of human placental development.

## Experimental procedures

### Key resources

Key resources for this study are listed in Table 1.

**Table 1:** Key resources

Reagent/Resource	Source	Identifier
hTSC cell lines		
T1, T2 hTSCs	This study; primary CTBs from Amnion Foundation	N/A
CT 29 hTSCs	Okae *et al.* (5)	RRID: CVCL_A7BA
CT 30 hTSCs	Okae *et al.* (5)	RRID: CVCL_A7BA
Antibodies		
Anti-KRT7	Cell Signaling Technologies	Cat #4465, RRID: AB_11178382
Anti-hCG	Abcam	Cat #ab9582, RRID: AB_296507
Anti-p63	Cell Signaling Technologies	Cat #13109, RRID: AB_2637091
Anti-TEAD4	Abcam	Cat #ab58310, RRID: AB_945789
Anti-CDX2	Abcam	Cat #ab76541, RRID: AB_1523334
Anti-GATA3	Cell Signaling Technologies	Cat #5852, RRID: AB_10835690
Anti-YAP	Cell Signaling Technologies	Cat #14074, RRID: AB_2650491
Anti-TFAP2C	Cell Signaling Technologies	Cat #2320, RRID: AB_2202287
Anti-Notch1	Cell Signaling Technologies	Cat #4380, RRID: AB_10691684
Anti-HLA-G	Abcam	Cat #ab52455, RRID: AB_880552
Anti-SDC1	Abcam	Cat #sc-50369, RRID: AB_2101536
Rabbit monoclonal IgG	Abcam	Cat #ab172730, RRID: AB_2687931
Rabbit XP IgG	Cell Signaling Technologies	Cat #3900, RRID: AB_1550038
Mouse IgG1	Abcam	Cat #ab18447, RRID: AB_2722536
Mouse IgG2a	Abcam	Cat #554126, RRID: AB_479661
Mouse IgG2b	Sigma	Cat #MABC006, RRID: AB_97848
Alexa Fluor plus 488–conjugated anti-rabbit IgG	Thermo Fisher Scientific	Cat #A32731, RRID: AB_2633280
Alexa Fluor plus 647–conjugated anti-rabbit IgG	Thermo Fisher Scientific	Cat#A32728, RRID: AB_2633277
DAPI	R&D Systems	Cat#5748
Chemicals, peptides, and recombinant proteins		
TrypLE Express	Thermo Fisher Scientific	Cat #12604013
Vitronectin	Thermo Fisher Scientific	Cat #A14700
Laminin-521	Stem Cell Technologies	Cat #77003
SB431542	Tocris	Cat #1614
Y-27632 dihydrochloride	Tocris	Cat #1254
CHIR99021	Tocris	Cat #4423
EGF	Stem Cell Technologies	Cat #78006.2
35 mm polystyrene, TC-treated Petri dish	Sarstedt	Cat #83.3900
CELLview glass plates	Greiner Bio-One	Cat #627965
24-well No. 1.5 coverslip 13 mm glass diameter	MatTek	Cat #P24G-1.5-13-F
4% Paraformaldehyde in PBS	Thermo Fisher Scientific	Cat #R37814
Triton X-100	Sigma	Cat #T8787
PBS w/o Ca/Mg	Sigma	Cat #D5773
PBS w/Ca/Mg	Sigma	Cat #D8662
Human IgG	Immunoreagents	Cat #Hu-003-C
BSA	Thermo Fisher Scientific	Cat #BP9703
10% BSA fatty acid free in PBS	Sigma	Cat #A1595
VPA	Sigma	Cat #P6273
A83–01	Tocris	Cat #2939
2-Mercaptoethanol	Sigma	Cat #M3148
FBS	Thermo Fisher Scientific	Cat #16141-061
DMEM/F12	Thermo Fisher Scientific	Cat #11320033
ITS-X	PeproTech	Cat #00101
L-ascorbic acid	Sigma	Cat #A8960
Penicillin-Streptomycin	Thermo Fisher Scientific	Cat #15140122
Laminin-111	R and D Systems	Cat #3446005-01
Di-8-ANEPPS	Thermo Fisher Scientific	Cat #D3167
RNeasy Mini Kit	Qiagen	Cat #74134
FluoroBrite^TM^ DMEM	Thermo Fisher Scientific	Cat #A1896701
AlbuMAX^TM^ II lipid-rich BSA	Thermo Fisher Scientific	Cat #11021029
Chemically defined lipid concentrate	Thermo Fisher Scientific	Cat #11905031
Oleoyl-L-α-lysophosphatidic acid sodium salt	Sigma	Cat #L7260
UK5099	Tocris	Cat #4186
NRG1	Cell Signaling Technologies	Cat #5218
Forskolin	Tocris	Cat #1099
KnockOut serum replacement	Thermo Fisher Scientific	Cat #10828028
Matrigel^TM^	Corning	Cat #356237
Software and algorithms		
Fiji	https://imagej.net/software/fiji/downloads	N/A
Zeiss Zen Software	https://www.zeiss.com/microscopy/us/products/microscope-software/zen-lite.html	N/A
MATLAB	https://www.mathworks.com/downloads	N/A
R and R studio	https://cran.r-project.org/bin/ https://posit.co/downloads/	N/A

BSA, bovine serum albumin; CTB, cytotrophoblast; DMEM, Dulbecco's modified Eagle medium; hTSC, human trophoblast stem cell; ITS-X, insulin-transferrin-selenium-ethanolamine; VPA, valproic acid.

### Derivation and culture of hTSCs

CTBs from two deidentified term placentas with no known pathology were provided by Amnion Foundation on a fee-for-service basis. Briefly, placental CTBs were enriched from placental tissue by Percoll gradient centrifugation and CD49f+ (*i.e.*, integrin α6+) cells were subsequently isolated using standard protocols and frozen in aliquots of 500,000 cells each. Frozen aliquots of CTBs received from Amnion Foundation were thawed directly into wells of a 24-well plate precoated with 3 μg/ml of vitronectin and 1 μg/ml of laminin-521, in TUA medium. TUA medium is TSCM described by Okae *et al.* (5) (Dulbecco's modified Eagle medium/nutrient mixture F-12 (DMEM/F-12) supplemented with 0.1 mM 2-mercaptoethanol, 0.2% FBS, 0.5% Penicillin-Streptomycin, 0.3% BSA, 1% insulin-transferrin-selenium-ethanolamine (ITS-X), 1.5 μg/ml L-ascorbic acid, 50 ng/ml EGF, 2 μM CHIR99021, 0.5 μM A83-01, 1 μM SB431542, 0.8 mM VPA, and 5 μM Y-27632), supplemented with 50 nM UK5099 and 0.2% AlbuMAX II. Cells were cultured in 5% CO_2_ at 37 °C and medium was changed every other day. Initially, very slow cell growth was observed. Cells were passaged using treatment with TrypLE Express for 10 to 15 min at 37 °C once cells became confluent or after 14 days, whichever was sooner; initial split ratios were 1:1 to 1:2. Subsequently, cells were transferred to 35 mm plates and routinely passaged every 7 to 10 days at a typical split ratio of 1:10. In some experiments, primary CTBs were also thawed directly into TSCM, TA medium (TSCM+0.2% AlbuMAX II), TUL medium (TSCM+50 nM UK5099+10 nM LPA), or TUC medium (TSCM+50 nM UK5099+0.1% CDL). After five passages, T1 and T2 hTSCs derived in TUA medium were additionally transitioned into TSCM. T1 and T2 hTSCs, as well as CT29 and CT30 hTSCs derived from first-trimester CTBs were cultured in TSCM as previously described (15).

### EVT and STB differentiation

EVT and STB differentiation was carried out using protocols previously described by Karakis *et al.* (15) or Okae *et al.* (5). Prior to differentiation, hTSCs at confluence were dissociated into single cells using TrypLE Express, and cells were seeded into wells of a 24 well glass-bottom plate (∼45,000) or 35 mm glass-bottom plates; plates were precoated with 3 μg/ml of vitronectin and 1 μg/ml of laminin-521. Studies using the protocol by Karakis *et al.* were conducted as follows: For STB differentiation, cells were seeded in defined trophoblast differentiation medium (DTDM; DMEM/F-12 with 1% ITS-X, 75 μg/ml L-ascorbic acid) supplemented with 50 ng/ml EGF and 5 μM Y-27632 at passage. On day 2 and day 4, medium was replaced with DTDM. Cells were fixed on day 6. For EVT differentiation, cells were seeded in DTDM supplemented with 50 ng/ml EGF, 5 μM Y-27632, and 150 μg/ml laminin-111. On day 2and day 4, medium was replaced with DTDM; cultures were analyzed on day 6. Studies using the protocol by Okae *et al.* were conducted as follows: For STB differentiation, cells were seeded in STB medium (DMEM/F12 supplemented with 0.1 mM 2-mercaptoethanol, 0.5% Penicillin-Streptomycin, 0.3% BSA, 1% ITS-X supplement, 2.5 μM Y-27632, 2 μM forskolin, and 4% KnockOut serum replacement). Medium was replaced on day 3 and cells were fixed on day 6. For EVT differentiation, cells were seeded in cultured in EVT medium (DMEM/F12 supplemented with 0.1 mM 2-mercaptoethanol, 0.5% Penicillin-Streptomycin, 0.3% BSA, 1% ITS-X supplement, 100 ng/ml NRG1, 7.5 μM A83-01, 2.5 μM Y27632, and 4% KnockOut serum replacement); Matrigel was added to a final media concentration of 2% after suspending the cells in EVT medium. On day 3, the medium was replaced with the EVT medium without NRG1 and Matrigel was added to a final concentration of 0.5%. Cells were fixed on day 6.

### Immunostaining

Cells were fixed with 4% paraformaldehyde for 10 min, permeabilized with 0.5% Triton X-100 in PBS for 10 min, and subsequently incubated in blocking buffer (0.5% BSA, and 200 μM human IgG in PBS) for at least 1 h. Cells were then incubated overnight at 4 °C on a rocker with primary antibodies diluted in blocking buffer. Primary antibodies used were anti-HLA-G (1:250), anti-Notch1 (1:250), anti-hCG (1:50), anti-SDC-1 (1:500), anti-KRT7 (1:50), anti-p63 (1:50), anti-TEAD4 (1:50), anti-CDX2 (1:250), anti-GATA3 (1:500), anti-YAP (1:200), and anti-TFAP2C (1:400). Corresponding isotype controls (rabbit monoclonal IgG, rabbit XP IgG, mouse IgG1, mouse IgG2a, and mouse IgG2b) were used at the same concentrations as the primary antibodies. Subsequently, cells were washed twice with PBS and labeled with secondary antibodies (Alexa Fluor 488 rabbit– or Alexa Fluor 647 mouse–conjugated antibodies) and 4prime,6-diamidino-2-phenylindole (DAPI) in blocking buffer for 0.5 to 1 h. Finally, cells were washed twice in PBS and stored in PBS prior to imaging with a laser scanning confocal microscope (LSM880, Carl Zeiss).

### Confocal microscopy image analysis

Image analysis was conducted using an image processing algorithm created in MATLAB R2021a, as previously described (15, 31). Briefly, TIFF images were generated after background correction in Zeiss Zen Lite software, using settings for corresponding isotype images for all samples. The algorithm identifies cells using DAPI staining (blue channel) and determines relative average protein expression intensity corresponding to antibody staining by mapping the red/green channels to individual cells based on the blue channel. For analysis of nuclear expression, only green/red pixels overlapping with the blue/pixels were considered. Expression intensity was set to zero if the average intensity is lower than the average intensity of all cells in the isotype control image. Data were normalized by the average isotype control expression intensity. Analysis for each experimental condition used 14 images (7 from each of two biological replicates); seven images were used for isotype controls. This was performed for 7 isotype control images and 14 experimental images (7 images for each of two replicates). Statistical analysis was conducted using the nonparametric Mann–Whitney *U* test, in Microsoft Excel (https://github.com/mjabn/-Mann-Whitney-U-test) as described (15). Results of this test are given as a *p* value to compare differences in medians. Statistical significance was inferred at *p* < 0.05.

### Membrane staining and calculation of fusion index for STB differentiation

Cells were washed with PBS (with Mg^2+^ and Ca^2+^) and incubated with 1 to 2 μM Di-8-ANEPPS and DAPI in PBS (with Mg^2+^ and Ca^2+^) on ice for ∼0.5 to 1 h. Subsequently, cells were washed once with FluoroBrite DMEM and imaged in FluoroBrite DMEM or PBS (with Mg^2+^ and Ca^2+^) using a Keyence BZ-X810 system. The fusion index for each condition was calculated using two biological replicates per cell line, except for T2 hTSCs in TUA medium differentiated using the protocol by Karakis *et al.*, where a single replicate was used. A total of seven images were captured for each replicate. Single cells and fused cells were individually quantified from each image, and the number of nuclei in each fused cell was determined. The fusion index for a single replicate was calculated as (N-S)/T, where N is the number of nuclei in syncytia, S is the number of syncytia, and T is total count of nuclei in both fused and unfused cells from seven images. Fused cells with n ≥ 3 nuclei were considered as syncytia.

### Total RNA extraction and sequencing

RNA was isolated using Purelink RNA Mini Kit using the manufacturer’s protocol and quantified using a Nanodrop 1000 spectrophotometer (Thermo Fisher Scientific). RNA samples were collected from at least three independent replicates, at different passage numbers. Library preparation with polyA selection and Illumina sequencing was conducted at Azenta Life Sciences as follows. RNA samples were quantified using Qubit 2.0 Fluorometer (Life Technologies), and RNA integrity was checked using Agilent TapeStation 4200 (Agilent Technologies). RNA-Seq libraries were prepared using the NEBNext Ultra II RNA Library Prep Kit for Illumina using manufacturer’s instructions (NEB). Briefly, mRNAs were initially enriched with oligo-dT beads. Enriched mRNAs were fragmented for 15 min at 94 °C. First strand and second strand cDNA were subsequently synthesized. cDNA fragments were end repaired and adenylated at 3′ ends, and universal adapters were ligated to cDNA fragments, followed by index addition and library enrichment by PCR with limited cycles. The sequencing library was validated on the Agilent TapeStation (Agilent Technologies), and quantified by using Qubit 2.0 Fluorometer as well as by quantitative PCR (KAPA Biosystems). The sequencing libraries were multiplexed and clustered onto a flowcell on the Illumina HiSeq 4000 instrument according to manufacturer’s instructions. The samples were sequenced using a 2 × 150 bp paired-end configuration. Image analysis and base calling were conducted by the NovaSeq Control Software (https://support.illumina.com/downloads/novaseq-control-software-v1-8.html). Raw sequence data (.bcl files) generated from Illumina HiSeq was converted into FASTQ files and demultiplexed using Illumina bcl2fastq 2.20 software (https://support.illumina.com/downloads/bcl2fastq-conversion-software-v2-20.html). One mismatch was allowed for index sequence identification.

### Analysis of RNA-Seq data

Raw FASTQ formatted sequence reads were imported into CLC Genomics Workbench (version 23.0.2 and 25.0, QIAGEN Digital Insights). The imported reads were trimmed based on quality score and ambiguous nucleotides were also removed using default software settings. The trimmed sequences were mapped to the reference genome, GRCh38.110 using the RNA-Seq analysis feature in CLC Genomics Workbench; default mapping and expression settings were used. For PCA and DEG analysis, “PCA for RNA-Seq” and “Differential Expression for RNA-Seq” toolsets were used, respectively. For heat maps, Euclidean distance with complete linkage option was used; a fixed number of 10,000 features, with five or more counts in at least one sample were used as filtering options. The heat maps were clustered based on metadata groups. For targeted gene analysis, |log_2_ (fold change)| ≥1 in expression between comparison groups with a threshold FDR-adjusted *p* value < 0.05 was used. The following datasets from the literature were used for comparative analysis: CT29 and CT30 hTSCs in TSCM (8) (GSE137295); hTSCs derived from term CTB in TSCM at 1% oxygen concentration (14) (GSE158901); primary vCTBs from the first-trimester (6) (GSE109976). Additionally, the following datasets were used for comparative analysis with hTSCs from hPSCs: from Dong *et al.* (9) (GSE138762), we included GSM4116158 (H9 naïve TSC Bulk RNA-Seq) and GSM4116160 (WIBR3 naïve TSC Bulk RNA-Seq). From Guo *et al.* (22) (GSE167089), we included GSM5092604, GSM5092605 (hNES1), GSM5092608, GSM5092609 (niPSC2), and GSM5092612, GSM5092613 (niPSC4). From Io *et al.* (23) (GSE144994), we included GSM4304002, GSM4304003, GSM4304004, GSM4304005, GSM4304006, and GSM4304007. From Zorzan *et al.* (24) (GSE184562), we included GSM6754200, GSM6754201, and GSM6754202, representing replicates of ccTSC.02. From Mischler *et al.* (8) (GSE137295), we included GSM4074861, GSM4074862, and GSM4074863 (hTSCs from H9 hESCs in TSCM), and GSM4074865, GSM4074866, and GSM4074867 (hTSCs from H1 hESCs in TSCM). Unless otherwise specified, first-trimester hTSCs, CT 29 and CT 30 were used as the control group for analysis.

The list of DEGs for various conditions was imported into IPA (Fall 2023 release version, QIAGEN Digital Insights) to analyze upregulated and downregulated biological pathways. A more stringent parameter list was used for IPA to identify the most abundant pathways across conditions; filtering parameters were as follows: FDR *p* value ≤0.05, Max group mean ≥ 5, |log_2_ fold change| ≥ 2. Statistical |Z-score| cutoff was set at 1 with a −log_10_ *p* value cutoff = 1.3 (*p* value = 0.05).

#### Cluster analysis and GO for analysis of RNA-Seq data

K-means cluster analysis was performed using R version 4.3.1 and R studio. Data of DEGs for different derivation conditions for hTSCs from term CTBs were collected from CLC genomic workbench and imported into R. The transcripts per million (TPMs) values for these genes from multiple replicates were averaged and used for pairwise analysis. CT29 and CT30 TPM values were used as control in this pairwise analysis for these three term-derived hTSC conditions. Data processing involved removing missing values, assigning gene names as identifiers, and log2 transformation of TPM values to normalize the data. The optimal number of clusters (k) was determined using the Elbow method, which plots the total within-cluster sum of squares *versus* k values (from 2 to 10 in this analysis). The sharpest decline in the plot determines optimal k value. We found k value equal to 3 for TUA and TSCM f TUA. But for 1% oxygen condition, k value was equal to 4.

Following clustering, GO analysis was performed separately for each gene cluster to identify enriched biological processes. Gene lists from each cluster were extracted and subjected to GO enrichment analysis. We used clusterprofiler package in R. The analysis focused on the biological processes category. Default *p* value and q value were used for enrichGO function. The full list of GO enrichment results is available in Tables S7–S19. The code used for this cluster and GO analysis is available at https://github.com/mjabn/K-means-clustering.

## Data availability

RNA-Seq data generated in this study have been deposited in Gene Expression Omnibus (accession number GSE267112). Additionally, RNA-Seq data from the literature used in this study are available in Gene Expression Omnibus (accession numbers GSE137295, GSE158901, GSE109976, GSE138762, GSE167089, GSE144994, GSE184562, and GSE137295). The code used for image analysis can be found at https://doi.org/10.5281/zenodo.7700524. All other data are available within the article and its supplementary materials.

## Supporting information

This article contains supporting information. Supporting information includes 9 Supplementary figures and 29 Supplementary tables (15).

## Conflict of interest

The authors declare that they have no conflicts of interest with the contents of this article.
